# Drought Tolerance in Plants: Physiological and Molecular Responses

**DOI:** 10.3390/plants13212962

**Published:** 2024-10-23

**Authors:** Mostafa Haghpanah, Seyyedhamidreza Hashemipetroudi, Ahmad Arzani, Fabrizio Araniti

**Affiliations:** 1 Kohgiluyeh and Boyer-Ahmad Agricultural and Natural Resources Research and Education Center, Dryland Agricultural Research Institute, AREEO, Gachsaran 7589172050, Iran; masoudhgh@gmail.com; 2 Genetics and Agricultural Biotechnology Institute of Tabarestan (GABIT), Sari Agricultural Sciences and Natural Resources University, P.O. Box 578, Sari 4818166996, Iran; irahamidreza@yahoo.com; 3 Department of Agronomy and Plant Breeding, College of Agriculture, Isfahan University of Technology, Isfahan 8415683111, Iran; 4 Department of Agricultural and Environmental Sciences—Production, Landscape, Agroenergy, University of Milan, 20133 Milan, Italy

**Keywords:** dehydration, drought stress, dry weather, osmotic stress, water deficit

## Abstract

Drought, a significant environmental challenge, presents a substantial risk to worldwide agriculture and the security of food supplies. In response, plants can perceive stimuli from their environment and activate defense pathways via various modulating networks to cope with stress. Drought tolerance, a multifaceted attribute, can be dissected into distinct contributing mechanisms and factors. Osmotic stress, dehydration stress, dysfunction of plasma and endosome membranes, loss of cellular turgidity, inhibition of metabolite synthesis, cellular energy depletion, impaired chloroplast function, and oxidative stress are among the most critical consequences of drought on plant cells. Understanding the intricate interplay of these physiological and molecular responses provides insights into the adaptive strategies plants employ to navigate through drought stress. Plant cells express various mechanisms to withstand and reverse the cellular effects of drought stress. These mechanisms include osmotic adjustment to preserve cellular turgor, synthesis of protective proteins like dehydrins, and triggering antioxidant systems to counterbalance oxidative stress. A better understanding of drought tolerance is crucial for devising specific methods to improve crop resilience and promote sustainable agricultural practices in environments with limited water resources. This review explores the physiological and molecular responses employed by plants to address the challenges of drought stress.

## 1. Introduction

Drought stress results from scarce water supply, triggering molecular, biochemical, and physiological alterations intended at adapting to the water deficit. Drought stress is one of the most significant abiotic stresses, adversely affecting plant growth and development. The depletion of water resources and the effects of climate change exacerbate the agricultural impact of drought [1]. Various factors contributing to the unpredictable nature of drought include the rhizosphere’s ability to retain water, evapotranspiration, and unreliable and uneven precipitation [2]. A large portion of plant canopy biomass is composed of water, which plays a crucial role in various physiological processes essential for plant metabolism, growth, and development. As a result, the dry season can be the most critical and stressful period for plant growth, particularly in regions prone to drought [3]. This has led to significant famines in the past and remains one of the greatest threats to global food security in the future [4].

Drought stress at any stage of plant growth can have detrimental effects on crop growth and development. However, the extent of damage depends on the severity of the stress, the crop species, and the specific growth stage. Drought stress adversely impacts nearly all features of plant metabolism, leading to changes in morphology, physiology, biochemistry, and molecular processes. In the following sections, we first discuss the effects of drought stress on plants.

This review aims to integrate current information on the physiological and molecular responses of plants to drought stress. We set out to provide an in-depth overview of signaling pathways, genetic components, and metabolic changes involved in drought response. This study also seeks to identify gaps in the current research and suggest potential avenues for further research, with the ultimate objective of contributing to the development of improved crop varieties and better agricultural decisions amidst increasingly unfavourable climate conditions.

## 2. Effects of Drought Stress

### 2.1. Cellular Effects of Drought Stress

**Figure 1:**
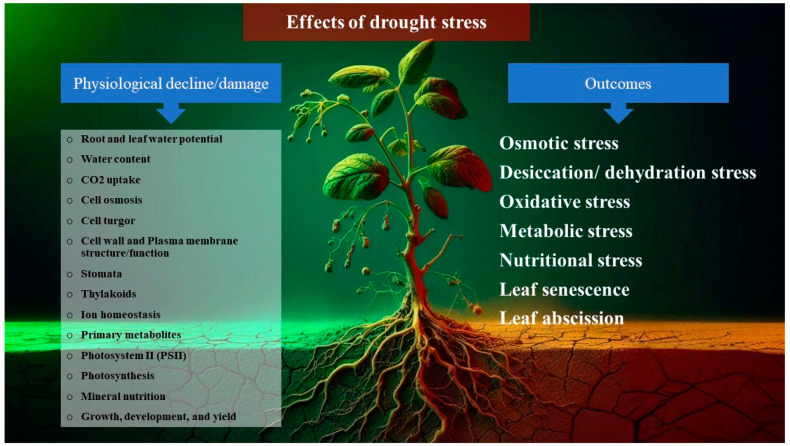
Physiological effects of drought stress on plants and the outcomes that cause growth and yield reduction.

The most important consequences of drought stress are osmotic stress, dehydration stress, dysfunction of plasma and endosome membranes, loss of cellular turgidity, inhibition of metabolite synthesis, cellular energy depletion, impaired photosynthesis, and oxidative stress (Figure 1). The cellular effects of desiccation have garnered significant interest over the years, yet there is still no consensus on how they function. However, the loss of cell membrane integrity is believed to be one of the primary causes of cell death following exposure to severe drought stress, leading to desiccation. Plant cells express various proteins to withstand and reverse the cellular effects of drought stress. Water deficit diminishes the hydrophobic effect, resulting in protein denaturation and a membrane phase transition from lamellar to inverted hexagonal [5].

Additionally, water loss causes the endosomal and plasma membranes to come closer together, facilitating lipid exchange and altering membrane permeability [6]. This triggers a chain reaction involving ion influx, increased levels of reactive oxygen species (ROS), and oxidative damage to lipids, nucleic acids, and proteins, which could ultimately lead to cell death. The lipids composing the membranes of mitochondria make this organelle one of the most vulnerable cellular sites to oxidative stress.

Oxidative stress damages cells and their subcellular components by inactivating enzymes, disintegrating membranes, and damaging cellular organelles such as mitochondria and chloroplasts in plants [7]. Dehydration resulting from a cellular water deficit also damages membranes and membrane lipids. Membrane lipid composition is associated strongly with membrane fluidity and stability under drought-stress conditions. Phosphatidylethanolamine (PE), phosphatidylcholine (PC), and phosphatidylglycerol (PG) are primary degradation targets among the principal phospholipids. A drastic reduction in galactolipids and phospholipids (PE, PC, and PG) occurs in plants suffering from water deficit stress, including the photosynthetic chloroplast membranes in higher plants [8]. Additionally, part of the membrane disruption results from increased free radicals and reactive oxygen species leading to membrane degradation through lipid peroxidation [9].

### 2.2. Effects of Drought Stress on the Structure and Function of Photosynthetic Apparatus

Photosynthesis progressively decreases during drought, but the mechanistic basis for this reduction remains somewhat debatable. In C3 plant species, stomatal limitations significantly contribute to the decline in photosynthesis during drought conditions, while in C4 plants, this decline is primarily due to metabolic constraints on CO_2_ assimilation [10]. Nonetheless, both stomatal limitations under mild to moderate drought conditions and non-stomatal factors under severe drought conditions may significantly reduce C3 photosynthesis [11,12]. Stomatal closure due to water stress decreases CO_2_ influx, thereby limiting photosynthesis. Consequently, the concentration of CO_2_ in the chloroplast stroma decreases, resulting in photorespiration. This process involves the enzyme Ribulose-1,5-bisphosphate carboxylase/oxygenase (Rubisco) catalyzing the reaction of oxygen with Ribulose-1,5-bisphosphate (RuBP), which leads to a loss of photosynthetic energy [13]. Drought causes a reorganization of the thylakoid membrane, particularly impacting the stacking of grana and reducing their number and layers [14]. Drought disrupts photosystem I (PSI) and photosystem II (PSII) at various stages of plant growth. The operational quantum efficiency of PSII (ΦPSII) is particularly sensitive to drought and can be used as an early indicator of drought stress [15]. Drought stress also impacts the function of vital photosynthetic enzymes, like Rubisco, resulting in a diminished photosynthesis rate [16]. Stress-tolerant switchgrass [17] and wild millet [18] genotypes, both C4 grass species, exhibited less reduction in photosynthesis (*A*) and stomatal conductance (*gs*) compared to stress-sensitive genotypes under drought stress conditions.

## 3. Drought Tolerance Mechanisms

**Figure 2:**
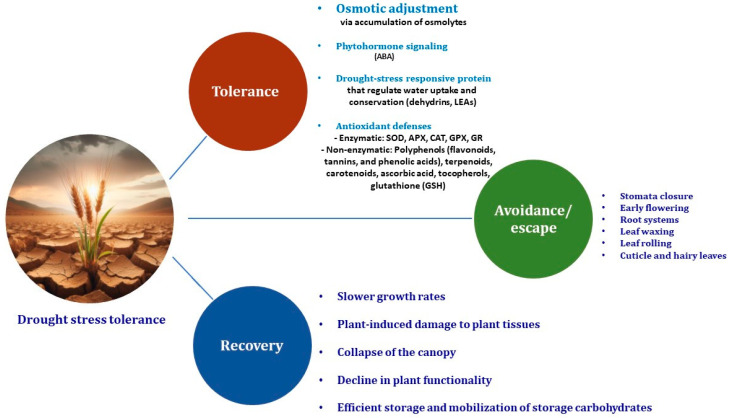
Plants employ various strategies, such as tolerance, recovery, avoidance, and escape, to cope with drought.

Understanding drought tolerance mechanisms is essential for developing adapted cultivars in sustainable crop production, especially given the increasing frequency of drought events caused by climate change. Drought stress adversely affects crops, reducing agricultural output and income for farmers [19]. Various strategies, such as recovery, avoidance, tolerance, and escape, are employed by plants to cope with drought (Figure 2), and these mechanisms are essential for improving crop stress resilience and plasticity [20]. Nevertheless, it is crucial to note that some mechanisms evolved in wild plants to improve drought tolerance may lead to reduced seed yield, highlighting the need for a cautious approach to avoid limiting productivity [21]. Additionally, reconciling drought tolerance with crop yield remains a significant challenge, and alternative approaches, such as genetic and molecular strategies, are being explored to address this issue.

Drought resistance mechanisms in plants encompass four categories: recovery, avoidance, tolerance, and drought escape. These mechanisms involve physiological, biochemical, and genetic aspects that enable plants to endure severe dehydration, regulate growth periods to avoid moisture stress and sustain important physiological processes under mild drought. Additionally, developing crop plants that are better able to tolerate and recover from drought stress is essential for improving agricultural resilience and fitness. Furthermore, exploring drought-tolerance and adaptation mechanisms is vital for mitigating the detrimental impacts of drought stress on crop yield, including reduced water content, turgor pressure, gas exchange, and photosynthetic activity [22].

This article aims to delve into the effects of drought stress on plant cells and explore the molecular and physiological mechanisms that enable drought tolerance in plants. The paper aims to provide insights into the detrimental effects of drought and the various strategies plants employ to mitigate the negative impacts of drought stress, including stress avoidance, escape, and tolerance. The paper also seeks to highlight the importance of understanding drought tolerance mechanisms for the sustainability of crop production and ensuring food security. Studying plant drought tolerance mechanisms involves physiological, biochemical, and genetic aspects that enable plants to endure dehydration and regulate growth periods to avoid moisture stress. The paper seeks to offer a comprehensive knowledge of plants’ molecular and physiological responses to drought stress, which can be used to develop sustainable approaches to alleviate the detrimental impacts of drought on agricultural productivity.

## 4. Physiological Responses

The vital physiological reactions crucial for enduring drought stress can be categorized into (I) partial closure of stomata to decrease water loss via transpiration, (II) osmotic adjustment to preserve cellular turgor through accumulation of compatible solutes (osmoprotectants), (III) synthesis of protective proteins like dehydrins, and (IV) triggering antioxidant systems to counterbalance oxidative stress. The following elaborates on the mechanisms mentioned above.

### 4.1. Stomatal Responses and Gas Exchange

Stomata closure is a crucial mechanism plants employ to reduce transpiration and cope with drought stress. Stomata regulate gas exchange, including the uptake of CO_2_ for photosynthesis and the release of oxygen. Stomatal closure is a prevalent adaptive response to the onset of water-deficit conditions in plants. Plants respond to water deficit by limiting water loss through decreased epidermal conductance and stomatal closure [23]. The strategy of dehydration avoidance, exemplified by stomatal closure, diminishes water loss from leaves. However, this action also restricts CO_2_ uptake, generates reactive oxygen species (ROS), damages photosystem II (PSII), and inhibits photosynthesis, consequently decreasing crop yield. Nonetheless, this approach proves effective in plants enduring mild to moderate drought conditions of short duration [24]. It is supplemented by synthesizing and mobilizing the ABA phytohormone, which induces stomatal closure. Stomatal closure is guided by both passive (hydraulic-mediated) and active (ABA-mediated) mechanisms. In grapevine, for example, hydraulic signals can induce the closure of stomata, which is an ABA process [25]. This closure diminishes water loss via transpiration, helping the plant conserve water during drought stress [26]. Drought priming, a process that enhances drought tolerance, has improved stomatal behavior to drought stress response. Drought priming has been found to enhance drought tolerance by improving both stomata closure and reopening rates. This fine-tuning of the stomatal closure process hinges on the species of plant and the conditions under which the drought stress occurs [27]. Therefore, the regulation of stomatal closure is a vital component of plant stress resilience and fitness, enabling plants to minimize water loss and cope with drought conditions [4]. During water stress, plant stomata are closed to preserve water, which lessens the loss of water vapour from the leaf surface and lowers transpiration rates. This adaptive response leads to a decrease in photosynthesis, a greater intrinsic water-use efficiency (iWUE) at the leaf level, and a reduction in CO_2_ uptake. While this helps water conservation, it has implications for other physiological processes, such as plant photosynthesis and temperature regulation [28,29]. Stomatal closure can affect the leaf temperature regulation mechanism, as transpiration helps cool the plant. Reduced transpiration may lead to elevated leaf temperatures, influencing plant metabolism [26]. Research has shown that decreased stomatal conductance during drought stress reduces photosynthesis, and plants exposed to drier conditions alter their stomatal traits to optimize water use, leading to enhanced water-use efficiency (WUE) [30]. Additionally, genotypes with lower stomatal density are more conservative in their water use and drought-tolerant in rice, suggesting that stomatal density contributes to water conservation and tolerance to drought [31].

The stomatal closure limits CO_2_ entry, leading to a photosynthetic rate decline, which can impact overall plant growth and productivity. This stomatal closure under water stress also decreases conductance, leading to a decline in photosynthetic rate and a higher intrinsic iWUE at the leaf level [28]. Although this adaptive response helps plants conserve water, it has drawbacks for photosynthesis and overall productivity [31]. As a result, the closure of stomata during drought stress affects plant physiology, underscoring the complex trade-off between photosynthetic activity and water conservation [32].

Stomatal closure is regulated by complex signaling pathways, including hormonal signals such as ABA, and affected by environmental factors like the plant’s water status and atmospheric humidity. The regulation of stomatal status under water deficit is primarily mediated by ABA, whose accumulation in the leaf activates stomatal closure [25,33]. Cytosolic pH, ROS, NO, and free Ca^2+^ are other signals involved in stomatal closure [26]. Further research is needed to understand interactions among these signaling factors [26].

Knowledge of the physiological and molecular mechanisms behind stomatal closure during drought stress is crucial for developing crop cultivars tolerant to drought. Key genes associated with this process allow the development of genetically modified crops with resilience to water scarcity through enhanced WUE. Advancements in genomics, transcriptomics, proteomics, and metabolomics have played a significant role in unravelling the complexities of the mechanisms of action of osmotic adjustment, enabling the identification of key metabolites, proteins, and genes associated with drought tolerance. In addition, research has explored the interaction between osmotic adjustment and other mechanisms of water stress adaptation, such as stomatal regulation, hormonal signaling pathways, and antioxidant defense, supplying a more accurate knowledge of the interconnected response networks that plants utilize to persist under dry conditions [24]. Production of transgenic crops for resilience to water scarcity through enhanced WUE is a potential strategy for improving plant adaptation to drought stress. Therefore, understanding the physiological and molecular mechanisms that modulate stomatal closure during water deficit is crucial for developing crop cultivars adapted to drought and contributing to more productive and sustainable agriculture under dry conditions [26,34,35].

The significance of drought tolerance concerning stomata encompasses not only the closure of stomata but also their overall number. Research has shown that modulation of stomatal density and movement contributes significantly to the tolerance of crop plants to water-deficit stress. For instance, a study demonstrated that enhancing plant tolerance to drought stress was achieved by regulating stomatal movement, resulting in plants with more closed stomata [36]. Additionally, the stomatal responses of different cultivars under drought conditions have been linked to their drought regulation strategies, with variations in ROS responses and stomatal closure affecting their adaptation to drought [37]. Furthermore, it is well-documented that stomatal activity is strongly associated with drought tolerance. However, there is limited research on how variations in stomatal morphology and density influence crop tolerance to drought. Manipulating stomatal density has been shown to enhance drought tolerance without adversely affecting nutrient uptake [31]. Research has demonstrated that rice plants with decreased stomatal density conserve water and exhibit enhanced water-deficit tolerance [23,38]. Also, Lonbani and Arzani (2011) showed that drought-tolerant triticale cultivars have a lower number of stomata when compared to other triticale and wheat cultivars. The synchronization of these mechanisms, including the number and movement of stomata, is essential for ensuring plant survival in challenging environmental conditions [39].

### 4.2. Osmotic Adjustment

Osmotic adjustment is a critical adaptive mechanism that enables plants to survive and thrive in challenging environmental conditions, such as drought, by preventing water loss and maintaining cell rigidity. Drought-tolerant genotypes resist dehydration through osmotic adjustment, which helps maintain cell turgor [20]. One of the key adaptative mechanisms in response to water deficits is the accumulation of solutes in the cells. Evidence implies that osmotic adjustment enhances yields in crop plants grown under drought-prone environments [40]. The accumulation of solutes in the vacuoles is a reversible physiological process that helps buffer cytosolic solutes and supports metabolism. Plants can regulate turgor primarily by accumulating solutes and potentially through the elastic adjustment of cell membranes [41]. An evaluation of wheat landraces and local cultivars showed that leaf rolling can delay or prevent cell death and help maintain grain yield under water-stress conditions. Genotypes with greater osmotic adjustment yield more than those with lower osmotic adjustment [42]. The postulation of genes for synthesizing compatible solutes, such as proline [43,44], diverse sugars [45], and the signaling pathways that modulate their expression are among the key findings [46]. In addition, the role of transcription factors and other regulatory components in cellular osmotic adjustment has been investigated [47].

The significant role of omics technologies in unravelling the complexities of osmotic adjustment is evident. This includes identifying key genes, metabolites, and proteins involved in drought tolerance [48]. For instance, physiological and molecular procedures were used to study osmotic adjustment during pre- and post-anthesis drought in wheat [49]. A study reviewed the role of osmotic adjustment and accumulation of compatible solutes in the cells in tolerance to dehydration, emphasizing the importance of osmotic adjustment in supporting production under water stress in plants [50].

### 4.3. Accumulation of Osmolytes

When plants experience water deficit conditions and the risk of dehydration, cellular turgor pressure decreases, adversely affecting physiological processes. Plants aim to maintain cell turgor and prevent wilting under drought conditions by employing osmotic adjustment strategies [51]. Under mild drought, drought-tolerant genotypes reduce evaporative water loss by adjusting osmotic pressure through accumulating compatible solutes and sustaining important physiological activities [23,52]. The accumulation of solutes in plant cells in response to water deficits can effectively adjust the osmotic potential (Ψs), helping maintain turgor pressure, and prevent wilting. The reversible accumulation of solute in vacuoles also significantly influences drought tolerance. Therefore, when the compatible solutes accumulate within cells, including cytosolic vesicles and vacuoles, they facilitate osmotic adjustment, which is crucial for helping plants to endure severe dehydration and maintain cellular turgor under water-deficit conditions [53]. These small organic molecules, known as solutes, do not interfere with cellular functions or structures, even at high concentrations [54]. Common osmolytes found in plants include the following:

**Proline and other amino acids** Proline helps stabilize the structures of plant cells and proteins and also scavenges ROS [55,56]. Under dehydration conditions, proline accumulates due to the upregulation induction of its biosynthesis and the inhibition of its degradation. Proline is a glutamic-acid-derived amino acid. Proline is biosynthesized via delta (1)-pyrroline-5-carboxylate (*P5C*) in two consecutive reductions catalyzed by two enzymes encoded by P5C synthetase (*P5CS*) and P5C reductase (*P5CR*) genes [55]. Proline accumulation is often associated with stress tolerance, where higher accumulations occur in stress-tolerant than in stress-sensitive plant genotypes [56]. Exogenous application of proline alleviates the adverse effects of drought stress on the growth and productivity of crop plants [56]. While proline helps alleviate abiotic stress, such as drought and salinity, its role is particularly significant under drought-stress conditions.

Glycine betaine (GB) and β-alanine betaine (AB) are other quaternary amino acid derivatives that serve as cellular solutes [57,58]. Similarly, these osmoprotectants play a role in stabilizing cellular structures and proteins and protecting cells from oxidative damage through ROS scavenging [57]. Glycine betaine commonly accumulates in plants under drought-stress conditions [59]. The accumulation of these amino acids is regulated at the transcriptional level of regulation of the genes, such as *betA* for GB and *β-alanine* for AB, involved in their biosynthesis [54].

**Sugars** The accumulation of sugars is consistently triggered by drought, altering the source–sink relationship in plants [60]. Plants accumulate sugars such as sucrose, glucose, galactose, maltose, lactose, raffinose, and fructose, which contribute to cellular osmotic adjustment and serve as an energy source during periods of stress [61]. Sugars act as osmolytes to adjust osmotic potential and serve as osmoprotectants in plants [61]. The accumulation of sugars in the cytosol and other subcellular compartments is regulated at the transcriptional levels of the related genes, such as *SWEET*s [62]. The accumulation of osmolytes, such as sugars, polyamines, and other compatible solutes, plays a role in safeguarding cellular structures under water-deficit-stress conditions [61,63]. Exogenous application of sugars also improves drought tolerance by alleviating the adverse effects of water stress on the growth and development of plants [64].

**Polyols** Polyols or sugar alcohols, including mannitol, inositol, galactinol, erythritol, glycerol, pinitol, and sorbitol, may accumulate in higher plant cells in response to drought stress. Polyols play a significant role as compatible solutes in the osmotic adjustment of plant cells under drought-stress conditions [65]. Intracellular accumulation of polyols and its regulation at the transcriptional level occur in response to water stress, as grape berries exemplify [66]. The accumulation of these osmoprotectants is associated with drought tolerance, and their concentration is usually greater in stress-tolerant than in stress-sensitive plants. The accumulation of these solutes alleviates the cellular osmotic potential, conserves turgor pressure, and sustains essential cellular functions in limited water availability conditions

### 4.4. Antioxidant Defense Systems

Activating the antioxidant system is a crucial mechanism higher plants use to counteract the oxidative stress resulting from the secondary effects of drought [22]. Due to the wide-ranging effects of oxygen toxicity on plant cells, oxidative stress is characterized by a shift in the balance between pro-oxidants and antioxidants, favoring the pro-oxidants and leading to potential damage. The term ROS refers to both free radicals and their non-radical intermediates. Species containing one or more unpaired electrons are called free radicals, and this incomplete electron shell gives them high reactivity. In biological systems, free radicals can be generated from nitrogen and oxygen. During water deficit, the balance between pro-oxidants and antioxidants is disrupted, accumulating reactive nitrogen species (RNS) and ROS in plant cells [67]. ROS, such as superoxide radicals, hydroxyl radicals, and hydrogen peroxide, along with RNS, such as peroxynitrite and nitric oxide, have the potential to cause cellular harm by oxidizing biomolecules like proteins, lipids, and nucleic acids [68]. Plants have evolved intricate antioxidant defense systems to mitigate the harmful impacts of oxidative stress. The antioxidant defense system ensures adequate protection against oxidative stress via ROS detoxification, diminished lipid peroxidation in membranes, and inhibiting damage to proteins by delaying oxidation and repairing nucleic acid (DNA) damage [69]. The antioxidant system can be separated into two parts: non-enzymatic and enzymatic. The enzymatic antioxidants that are known for their roles in scavenging RNS and ROS can be named catalase (CAT), superoxide dismutase (SOD), glutathione peroxidase (GPX), peroxidase (POX), glutathione reductase (GR), and glutathione-S-transferase (GST) and ascorbate peroxidase (APX) [70]. The AsA-GSH cycle is a key antioxidant defense pathway in plant cells used to detoxify hydrogen peroxide (H_2_O_2_), employing nonenzymatic antioxidants such as glutathione (GSH) and ascorbic acid (AsA) as well [71].

Research has shown that drought-tolerant genotypes exhibit differential activity in their antioxidant defense systems, leading to lower accumulation of ROS and higher quantity of antioxidant enzymes such as superoxide dismutase (SOD), peroxidase (POD), catalase (CAT), when compared to drought-sensitive ones [63]. Additionally, the up-regulation of the antioxidant activity through retrograde signaling is a crucial process in the acclimation of plants to oxidative stress, contributing to the enhanced drought tolerance of certain plant species [67]. Furthermore, the role of exogenous low-dose hydrogen peroxide in alleviating drought stress and the activation of the protective machinery in revival plants have also been studied in the context of the antioxidant activity and tolerance to drought in plants [67].

#### 4.4.1. Enzymatic Antioxidants

**Superoxide dismutase (SOD)** The SOD antioxidant enzyme has a role in mitigating oxidative stress induced by drought in crop plants. Various reports have demonstrated the role of SOD in diverse crops under drought-stress conditions. A study on the effects of drought stress on triticale revealed that genotypes with higher SOD activity produced higher greater yields [72]. SOD helps plants remove superoxide radicals by converting them into oxygen and hydrogen peroxide under stress [4]. The effect of abiotic factors such as water stress, temperature, and salinity on the expression of eight SOD genes during seed germination in sesame plants was investigated. The study observed a correlation between the reduction in germination parameters and the decrease in expression of the SOD genes under these abiotic stresses [73].

**Catalase (CAT)** Catalase contributes to the breakdown of hydrogen peroxide (H_2_O_2_) into oxygen and water, lowering the concentrations of this potentially harmful molecule. The studies have investigated the role of CAT in various crops in response to drought stress. A survey reported that the accumulation of drought-induced H_2_O_2_ was associated with reductions in soil water content (SWC) in wheat. Leaf CAT activity and CO_2_ were notably increased only in response to severe drought when the SWC dropped below 20% [74]. The transcript amounts of *CAT1* and *CAT2* varied across the day/night cycle in plants grown under normal conditions, while drought decreased the amounts of their mRNAs [74]. The CAT activity was significantly reduced under water-deficit stress and increased upon re-watering at the initial stage of the stress [75]. A study on cotton reported a drought-induced increase in CAT activity led to improved yield when grown under water-deficit conditions in the field [76].

**Ascorbate peroxidase (APX) and peroxidases (POD)** These enzymes help detoxify hydrogen peroxide by utilizing reducing equivalents provided by antioxidants like GSH and ASA. The overexpression of the ascorbate peroxidase (*PpAPX*) gene from *Populus peroxisomal* in transgenic tobacco enhanced tolerance to drought [77]. Stromal ascorbate peroxidase (*OsAPX7*) has been shown to modulate tolerance to drought in rice [78]. APX is a crucial antioxidant enzyme within these scavenging systems, facilitating the H_2_O_2_ to H_2_O conversion by utilizing ascorbate as an electron donor [79]. Furthermore, APX has demonstrated the ability to alleviate drought stress in rice [78]. POD oxidizes various substrates using H_2_O_2_ and helps prevent the excessive H_2_O_2_ accumulation that occurs under stress conditions.

#### 4.4.2. Non-Enzymatic Antioxidants

Phytochemicals, commonly known as plant secondary metabolites, comprise diverse natural products. Among these compounds are groups that actively participate in plant adaptation to biotic and abiotic stress [80]. Numerous phytochemicals have been recognized as non-enzymatic antioxidants, signifying their role in mitigating oxidative stress without directly engaging in enzymatic reactions. Below are some prevalent classes of phytochemicals that function as non-enzymatic antioxidants:

**Phenolic compounds** Phenolic compounds originate from phenylpropanoid, alkaloid, isoprenoid, or fatty acid pathways. However, phenolic compounds are more commonly derived from the phenylpropanoid or shikimic acid pathway [81]. They contain benzene rings adorned with one or more hydroxyl substituents. Primary metabolites like erythrose-4-phosphate and phosphoenolpyruvate are involved in the initial biosynthesis of phenolics. The shikimic acid pathway yields l-phenylalanine which then participates in the phenylpropanoid pathway to form p-coumaroyl CoA, thus initiating the synthesis of compounds like stilbenes or flavonoids [81]. Phenolic compounds have many molecular and biochemical functions in the plant kingdom, such as antioxidant activity, free radical scavenging, signaling, mediating auxin transport, and plant defense [82]. The contribution of phenolic compounds in plant adaptation to drought stress is a well-researched topic due to their vital role in regulating plant development and stress tolerance mechanisms. They are secondary aromatic metabolites synthesized by plants to provide tolerance during adverse conditions. These compounds significantly regulate above- and below-ground defense mechanisms to protect plants against biotic and environmental factors [83].

Abiotic factors, such as drought, increase phenolic compound accumulation to counteract potential toxic effects. In tomatoes, it was reported that under water-deficit stress, the accumulation of kaempferol and quercetin increased to detoxify the toxic impact of H_2_O_2_ [84]. Similarly, Rezayian et al. (2018) reported that total phenols, flavonoids, and flavonols increased in *Brassica napus* L. plants under drought stress [85]. The increased gene expressions associated with the biosynthesis pathway of polyphenols cause the accumulation of phenolics in plants in response to drought stress. André et al. (2009) reported that water-deficit stress induces the expression of genes related to the polyphenol biosynthesis pathway (*PAL*, *C3H*, *HCT*, *CHI*, *CHS*, *DFR*, *F3H*, and *AN1*) in potato tubes [86]. Park et al. (2023) identified 24 differentially expressed genes (DEGs) associated with regulating the biosynthesis of phenylpropanoid genes under drought stress in *Ligularia fischeri*. Among these DEGs, upregulated flavone synthase and anthocyanin 5-O-glucosyltransferase were identified as potential drought-responsive genes, which could contribute to the generation of high concentrations of anthocyanins and flavones in response to drought stress in *L. fischer* [87].

**Flavonoids** A diverse group of polyphenolics, present in various plant tissues, are classified as flavonoids. They exhibit potent antioxidant activity and can effectively scavenge free radicals. Examples include quercetin, kaempferol, and catechins. Flavonoids play a significant role in enhancing plants’ tolerance to drought. Many studies have established the positive impacts of flavonoids on plant growth and stress resilience. For instance, research on faba beans revealed that the application of biochar and rhizobacteria significantly increased the levels of flavonoids, total phenols, and other stress-related compounds, thereby enhancing drought tolerance [88]. Similarly, a study on rice plants revealed that the over-accumulation of flavonoids improved tolerance to drought and UV radiation stress by mitigating the accumulation of ROS [89]. Furthermore, research on *Ficus deltoidea* Jack plants demonstrated that the application of H_2_O_2_ increased the accumulation of flavonoids and other stress-related metabolites, leading to improved growth and drought tolerance [90].

**Phenolic Acids** Phenolic acids are compounds with a phenol ring and a carboxylic acid group. They exhibit antioxidant activity and are found in fruits, vegetables, and whole grains. Examples include caffeic acid and ferulic acid. Phenolic acids are a type of plant secondary metabolites that are synthesized under various environmental stressors, such as drought. Recent findings have shown that phenolic acids improve fruit quality under abiotic stress conditions [91]. Phenolic acids are also associated with the biosynthesis of other stress-related metabolites, including flavonoids, proline, anthocyanins, unsaturated fatty acids, and antioxidants, which contribute to plant stress resilience [92,93]. Additionally, phenolic acids have been linked to increased antioxidant activity, which is important for plant stress protection [94]. Furthermore, a study on sweet basil plants demonstrated that foliar spraying with phenylalanine and tryptophane, which are precursors of phenolic compounds, increased the total phenolic content and antioxidant activity of the plants in response to water-deficit stress [95]. Therefore, phenolic acids contribute to plant resilience by enhancing antioxidant activity and are important for plant quality and stress protection. However, the role of phenolic acids in improving plant resilience to water-deficit stress is not as well studied as that of flavonoids.

**Tannins** Tannins are polyphenolic compounds that can bind and precipitate proteins. They are found in various plant tissues, such as fruits, nuts, and seeds. Tannins have antioxidant properties and contribute to the astringent taste of some foods. Tannins are a type of polyphenolic compound found in plants, and they have been shown to protect plants against oxidative stress induced by drought stress. Tannins, specifically condensed tannins (CTs), have been demonstrated to act as potential antioxidants, shielding against oxidative and cellular harm induced by drought stress [96]. The antioxidant properties of tannins help in alleviating the detrimental effects of ROS generated in response to stress, thereby protecting the plant from oxidative damage [96]. Moreover, tannins are inducible of enzymatic antioxidants, suggesting that they have broader functional capabilities in defense against diverse abiotic stress in plants [96]. Therefore, tannins, particularly condensed tannins, contribute to plant resilience by acting as antioxidants and protecting plants against oxidative harm triggered by drought stress. Generally, there are two main types of tannins: hydrolyzable tannins and condensed tannins (CTs). These two kinds of tannins help plants tolerate drought stress in the following ways:

I. Scavenging reactive oxygen species (ROS): Tannins act as antioxidants, scavenging ROS generated under stress conditions, which helps mitigate oxidative damage in poplar trees [96].
II. Protecting cellular components: Tannins help maintain cellular homeostasis and protect vital cellular components from harm, minimizing oxidative damage [89].
III. Enhancing stress-related metabolites: Tannins are involved in the biosynthesis of other stress-related metabolites, such as proline, anthocyanins, unsaturated fatty acids, and antioxidants, which further contribute to plant stress resilience [96].
IV. Signaling molecule modulators: Tannins contribute to the biosynthesis and functional roles of signaling molecules, including hydrogen peroxide, jasmonic acid, salicylic acid, abscisic acid, and ethylene, all of which are linked to stress response pathways [96].
V. Alleviating stress indicators: Tannins can help alleviate stress indicators, such as malondialdehyde (MDA) contents and hydrogen peroxide (H_2_O_2_), which are associated with oxidative stress [97].
VI. Enhancing antioxidant enzymes: Tannins can boost the activity of antioxidant enzymes, such as POD, CAT, SOD, APX, and GPX, which help plants cope with oxidative stress [97]. Generally, both hydrolyzable tannins and condensed tannins contribute to plant resilience by scavenging ROS, protecting cellular components, enhancing stress-related metabolites, modulating signaling molecules, alleviating stress indicators, and enhancing antioxidant enzymes.

**Terpenoids** Terpenoids are a large and diverse class of compounds derived from isoprene units. They include terpenes and steroids, and some terpenoids, such as tocopherols (vitamin E) and ubiquinone (coenzyme Q), act as antioxidants [98,99]. Drought stress alters the distribution of carbon between primary and secondary metabolites in Scots pine trees. Pyruvate, a crucial metabolite originating from primary metabolism, serves as a substrate in various secondary pathways, contributing to the formation of numerous BVOCs, including volatile terpenoids, isoprene, oxygenated compounds, benzenoids, and products of fatty acid oxidation [100]. The application of potassium has been shown to effectively alleviate the negative impacts of drought stress on plant growth. However, few studies have investigated its application on the medicinal plant *Salvia miltiorrhiza* L. Applying potassium during drought conditions not only led to a significant reduction in amino acid content but also enhanced the levels of phenolic acids and terpenoids in the roots [101].

Drought stress can affect the production and accumulation of terpenoids in plants. Some of these effects include the following: I. Altered root exudate compositions: Drought stress can result in an alteration of the composition of root exudates, with an increase in the relative abundance of terpenoids [102]. II. Changes in terpenoid emissions: Drought stress can impact the emissions of terpenoids from plant leaves. For example, gum rockrose (*Cistus ladanifer* L.) and cork oak (*Quercus suber* L.) showed decreased total terpenoid emissions in response to drought stress, with *C. ladanifer* emitting a large variety of compounds that strongly decreased in response to drought [103]. III. Accumulation of active phytochemicals: Drought stress can accumulate higher concentrations of active phytochemicals like alkaloids, tannins, and terpenoids in plant parts such as roots, leaves, stems, flowers, fruits, and seeds [102].

**Carotenoids** Carotenoids, classified as isoprenoid compounds, play a crucial role as precursors for various compounds. These pigments are accountable for imparting the vibrant yellow, orange, and red hues found in numerous fruits and vegetables. Beta-carotene, lutein, and zeaxanthin are carotenoids that function as antioxidants by eliminating free radicals and quenching singlet oxygen.

Carotenoids, as isoprenoid compounds, contribute to drought tolerance in plants. Drought stress can impact the production and accumulation of carotenoids in plants, leading to various responses. Carotenoids, such as β-carotene, can trigger defense mechanisms that enhance plant tolerance to drought stress. They can also regulate root growth, affecting cell elongation and division, and contribute to increased drought tolerance, independent of stomatal closure [104]. Drought stress may lead to an increase in the overall antioxidant activity in both leaves and developing grains of plants. Also, drought stress may result in the alterations of the composition of root exudates, with an enhancement of the relative abundance of carotenoids [105]. In summary, carotenoids contribute to a multifaceted role in plant drought stress, influencing antioxidant activity, root development, and root exudate metabolomes. Their contribution to plant resilience under drought conditions makes them a significant focus of research in understanding and mitigating the impacts of drought on plants.

**Alkaloids** Alkaloids are nitrogen-containing compounds with diverse biological activities. Some alkaloids, such as quinine and nicotine, have been reported to possess antioxidant properties. Some studies have shown that drought stress can accumulate higher concentrations of active phytochemicals like alkaloids in plant parts such as roots, leaves, stems, flowers, fruits, and seeds [102]. Additionally, some alkaloids, such as quinine and nicotine, have been reported to possess antioxidant properties [106]. Nevertheless, available information concerning the roles of alkaloids in plant drought stress is scarce, emphasizing the necessity for further research to grasp their potential contributions in this regard comprehensively.

#### 4.4.3. Other Non-Enzymatic Compounds

**Ascorbate (vitamin C)** Ascorbate is a direct scavenger of ROS and a cofactor for some antioxidant enzymes [105]. The potential applications of ascorbate produced under drought stress in the food industry are not extensively documented in the provided search results. However, studies have shown that transgenic tobacco plants overexpressing dehydroascorbate reductase (DHAR), an enzyme involved in the regeneration of ascorbate, exhibited higher levels of reduced ascorbate and improved tolerance to oxidative stress, including water-deficit stress [107]. Ascorbate (AsA) and glutathione (GSH) play crucial roles as low molecular weight antioxidants within plant cells. These components play vital roles in plant metabolism and contribute to tolerance against various abiotic stresses, including salinity, drought, cold, and heat stress. They regulate cellular levels of H_2_O_2_ and are associated with the AsA-GSH cycle to detoxify H_2_O_2_. The balance between the reduced and oxidized forms of GSH and AsA is critical for redox signaling and the activation of stress response mechanisms in plants experiencing stress. AsA and GSH play vital roles in redox signal transduction and protecting cellular function [108].

**Glutathione (GSH)** Glutathione is a major cellular antioxidant that plays a role in the reduction of hydrogen peroxide and other ROS [109]. GSH plays a significant role in mediating ABA signaling and regulating seed dormancy and adaptation to drought in plants. Research by Koramutla et al. (2021) has underscored the interplay between ABA and GSH in regulating seed dormancy, germination, stomatal closure, and water-deficit tolerance [109]. In mung beans, GSH has been shown to induce drought stress tolerance at the seedling stage by coordinating the methylglyoxal detoxification systems and antioxidant defense, thereby alleviating oxidative damage and reducing methylglyoxal toxicity [110]. Glutathione (GSH) contributes to plant drought stress response through its multifaceted roles in mediating ABA signaling, regulating seed dormancy, and enhancing drought tolerance. GSH is a major cellular antioxidant that participates in the reduction of hydrogen peroxide and other reactive oxygen species (ROS) [109]. Additionally, GSH has been shown to induce drought stress tolerance in mung bean seedlings by coordinating the antioxidant defense and methylglyoxal detoxification systems, thereby alleviating oxidative damage and reducing methylglyoxal toxicity. The pivotal function of GSH in maintaining redox homeostasis, detoxifying extra ROS, and regulating protein roles underscores its significance in plant survival under stress, particularly drought [109]. The coordinated roles of GSH in enhancing the antioxidant defense system, up-regulating the glyoxalase system, and modulating proline and water content contribute to the plant’s capability to tolerate water-deficit induced oxidative stress and methylglyoxal toxicity [109].

**Chelation of metals** Chelation of metals involves certain antioxidants that can bind to metal ions, preventing their involvement in reactions that produce ROS. However, the search results do not directly address the various chelation methods that could mitigate plant drought stress. Further research is warranted to enhance our understanding of the diverse chelation methods that could effectively mitigate plant drought stress.

## 5. Molecular Responses

**Figure 3:**
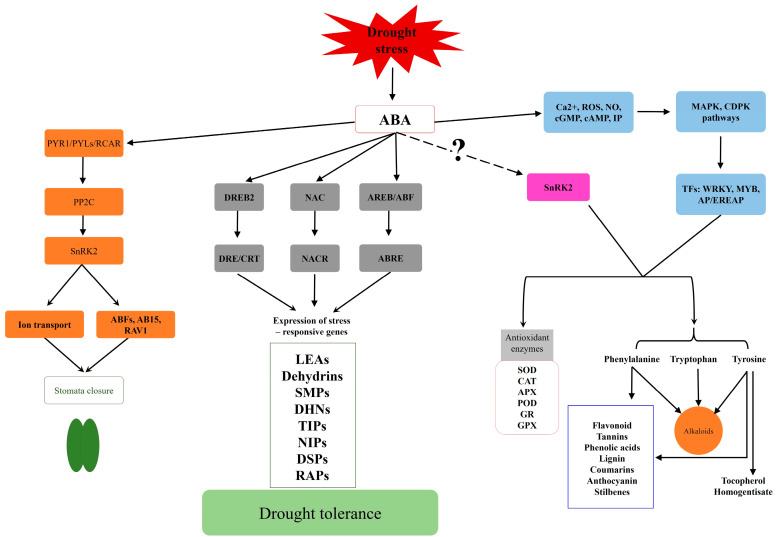
Molecular mechanisms of drought stress tolerance in plants. Abbreviations: ABA, abscisic acid; PYR1/PYL, pyrabactin resistance/PYR1-like; RCAR, Regulatory Component of ABA Receptor; PP2C, type 2C protein-phosphatase; SnRK2, sucrose non-fermenting 1-related protein kinase subfamily 2; ABF, ABA-responsive element binding factors; RAV1, Related to ABI3/VP1; DREB2, dehydration responsive element-binding protein 2; NAC, named based on its three domains: NAM, ATAF1,2 and CUC2; AREB, abscisic acid–responsive element binding protein; DRE/CRT, Dehydration Responsive Element/C-repeat Binding Factor; NACR, NAC recognition sequence; ABRE, ABA-responsive element; LEAs, Late embryogenesis-abundant; DHNs, Dehydrins, SMPs, seed maturation protein; TIPs, tonoplast intrinsic proteins; NIPs, nodulin 26-like intrinsic proteins, DSPs, desiccation-stress proteins, RAPs, resistance-associated proteins, Ca^2+^, calcium ion; ROS, reactive oxygen species; cGMP, 3’,5’-cyclic guanosine monophosphate; cAMP, cyclic adenosine monophosphate; MAPK, mitogen-activated protein kinase; CDPK, Calcium-dependent protein kinase; WRKY, WRKY transcription factors; MYB, MYB transcription factors; AP/EREAP, Activator protein/element of the apoptosis promoting; CAT, catalase; SOD, superoxide dismutase; APX, ascorbate peroxidase; POD, peroxidase; GR, glutathione reductase; GPX, Glutathione Peroxidase.

Enhancing drought tolerance in crop plants necessitates thoroughly comprehending the underlying molecular mechanisms [111]. Numerous genes contribute to drought tolerance, including those associated with signaling [112], transcription factors [113], and phytohormones [114]. Their activity may trigger genetic and epigenetic changes in gene expression, ultimately leading to the development of tolerance. Exploring these functional and molecular mechanisms in greater detail is essential for devising effective breeding strategies geared toward bolstering plant drought tolerance. The crucial molecular responses necessary for withstanding drought stress can be categorized at the three primary levels: I) induction of signaling genes, II) induction of transcription factors, and III) induction of stress-responsive genes (Figure 3).

These levels of regulation are parallel and interact with one another, most likely leading to the plant’s adaptation and survival in harsh environmental conditions via an integrated gene network. The induction of signaling genes, transcription factors and drought-stress-responsive genes will be briefly reviewed below.

### 5.1. Signaling Genes in Response to Drought

Drought triggers the expression of specific signaling genes that encode proteins known as receptors. These receptors can detect external cues and transmit that information to the cell, resulting in physiological and molecular responses [115]. Essential drought response signaling genes include:

**Mitogen-Activated Protein Kinase (MAPK)** The Mitogen-Activated Protein Kinase (MAPK) gene family is made up of serine/threonine protein kinases that play an important role in converting extracellular signals into intracellular responses [116]. This family features a conserved three-tiered phosphorylation cascade comprising three key elements: MAPK, MAPK Kinase (MAPKK), and MAPK Kinase Kinase (MAPKKK) [115]. Transcriptional factors that control gene expression in reaction to environmental stresses like drought are among the downstream targets activated when one component in the cascade phosphorylates and activates another. During drought stress, the MAPK cascade signaling module in cotton, comprising GhMAPKKKs3/8/31-GhMAPKK5-GhMAPK11/23, regulates transcription factor genes such as WRKYs, which are implicated in the transport pathways and synthesis of RALF, proline, and ABA [117].

**Calcium sensors** Calcium ions (Ca^2+^) serve as a prevalent second messenger in signal transduction pathways and regulate numerous molecular functions and biological processes in plants. The gene family involved in calcium-sensing comprises calcium-dependent protein kinases (*CDPKs*), calmodulin (*CaM*), calmodulin-like (*CML*), and calcineurin B-like proteins [118,119]. In response to drought stress, calcium sensors detect variations in intracellular calcium levels, activating calcium influx channels in the plasma membrane. Upon activation, these sensors convert the calcium signal into downstream actions, including the phosphorylation of target proteins that modulate the expression of genes associated with drought tolerance. Calcium sensors also play an important role in modulating stomatal closure by activating stress-responsive genes that enhance the expression of drought-associated genes [120]. They also interact with diverse signaling pathways, so incorporating multiple signaling systems boosts the plant’s tolerance to water deprivation.

**Receptor-Like Kinases (RLKs)** The ability of plants to communicate and become aware of their surroundings relies on receptors. Plants’ Receptor-Like Kinases (RLKs) use their extracellular, transmembrane, and intracellular domains to perceive environmental stimuli and initiate cascades of intracellular signaling events [121]. When RLKs detect drought signals, they activate certain signaling pathways that activate drought-responsive genes, such as those that regulate protective proteins and osmoprotectants [122]. Additionally, they are vital in controlling the closing of stomata, which reduces transpirational water loss. RLKs also work with other signaling pathways, including abscisic acid (ABA) signaling, to coordinate overall drought responses [123]. RLKs help plants reach deeper soil moisture and tolerate dryness by encouraging root growth.

### 5.2. Transcription Factors Involved in Drought Response

Three basic steps are often recognized in the molecular response to environmental changes, particularly drought stress: signal receipt, signal transduction, and expression of the set of genes involved in stress [124,125]. All these phases depend on transcription factors to regulate gene expression [126]. Proteins activating or inhibiting transcription and displaying sequence-specific DNA binding are known as transcription factors (TFs) [127]. Approximately plant genomes have more than 2000 genes that encode the TFs and are classified into 58 different families. As trans-acting factors, TFs interact directly or indirectly with cis-active elements to control the activation or deactivation of target gene expression [124]. Each TF binds to the matching motifs in the target genes’ proximal/distal promoter regions and regulates their expression based on its domain/motif [128,129]. Some TFs also play a role in the stress-induced activation or deactivation of downstream gene expression by acting as molecular switches in conjunction with other transcription complex regulatory proteins [130].

Discovering and studying gene families’ functions has been made feasible by the advent and use of next-generation sequencing (NGS) technology in the past ten years, as well as by the availability of the genome sequences of several non-model plants.

These developments have aided in the identification of the molecular role of these genes under biotic and abiotic stress conditions. Several studies have shown that certain families of transcription factors (TFs) play a crucial role in the regulatory network that controls how plants respond to drought stress [131,132]. These gene families, such as *WRKY*, *NAC*, *DREB*, *MYB*, and *bZIP*, play a crucial role in the stress response, and analyzing their expression profiles can enhance our understanding of the underlying molecular processes. Examining the expression profile of TF families may provide light on the molecular mechanisms underlying plants’ responses to drought stress and lead to strategies for developing drought resistance in plants.

The response of plants to drought stress is regulated by many families of transcription factors, according to recent findings [133]. Through the regulation of the expression of target genes, these factors enable plants to efficiently deal with the unfavorable circumstances that are present in their environment. As an example, recent research conducted by Qu, Zou [12] showed that the *OsFLP* transcription factor, which is a member of the *R2R3*-*MYB* family, positively regulates the *NAC* factor’s expression and hence the rice plant’s response to drought stress [134]. These findings emphasize the significance of interactions among various transcription factors in stress response signaling networks and facilitate the discovery of novel potential targets for enhancing plant resistance to water stress. Integrating transcriptomics and metabolomics data can also reveal drought-responsive transcription factors and lead to innovative genetic engineering strategies to build drought-tolerant plants [135]. In the face of climate change, these all-encompassing methods have the potential to greatly enhance plant performance.

Here, we will take a quick look at a few important families of transcription factors in order to better understand how they contribute to the regulatory network that plants use to react to drought stress. A better understanding of drought stress and its key transcription factors, *NAC* and DREB, will be provided.

***NAC*** Among the several plant transcription factor families, the *NAC* family is among the most extensive and crucial in dealing with drought and other environmental challenges [136]. The genes of this family have been identified in most plants, and they have been analyzed and identified genome-wide using bioinformatics techniques. By binding to the sequences of cis-acting elements that are located in the promoter regions of the target genes, these factors are able to regulate the expression of the target genes. For instance, in the case of rice, the *SNAC1* factor enhances the ability to withstand drought by stimulating the expression of genes associated with abscisic acid and osmolytes [137].

The number of *NAC* genes varies among different plant species. For instance, Arabidopsis has been shown to have 117 *NAC* genes, whilst rice has only been reported to have 151 *NAC* genes [138]. Although *NAC* family members play a significant part in plant responses to environmental stressors, each gene’s exact function may differ. For instance, the *ANAC096* factor in Arabidopsis enhances drought and salinity tolerance by modulating the expression of genes associated with ethylene and abscisic acid [139]. However, *SNAC1* plays a larger role in drought response in rice [140]. Furthermore, NAC factors in various plants may interact with various proteins and are engaged in various signaling cascades. In transgenic Arabidopsis, PwNAC11 forms interactions with the transcription factor *ABF3* and *DREB2A* to activate the expression of *ERD1*, as shown by Yu et al. (2021) [141]. Similarly, in rice, *SNAC1* interacts with proteins associated with abscisic acid, as previously noted. According to Liu et al. (2023), the *ZmNAC20* factor controls the expression of genes associated with osmolytes and antioxidants in maize, which leads to an increase in drought tolerance [142]. Overall, the number of members, expression patterns, functions, and protein interactions of the NAC family in response to environmental stresses vary significantly between plants, contributing to the diversity of plant responses to environmental stresses.

***DREB*** The DREB (Dehydration-Responsive Element Binding) subfamily is part of the larger AP2/ERF (APETALA2/Ethylene Response Factor) gene family, which controls plant responses to environmental stresses [143]. The AP2/ERF family is commonly divided into two main divisions. The AP2 subfamily comprises proteins possessing two AP2 domains. While these proteins do not directly participate in the response to environmental stimuli, they play a crucial role in regulating floral development and stress responses. Members of the ERF subfamily are transcription factors that respond to ethylene and regulate reactivity to both biotic and abiotic stresses [144].

The DREB subfamily is classified as part of the ERF subfamily. This subfamily of transcription factors was initially discovered in *Arabidopsis thaliana* L. It is especially activated in response to dehydration, drought, and cold stress, and it aids in controlling the expression of genes linked to drought tolerance [145]. From an evolutionary point of view, the *DREB* subfamily has been extensively investigated across a wide range of plant species, particularly in cereal crops such as wheat and rice [146]. This subfamily contains several gene members, each of which, despite significant sequence similarity, may exhibit unique features and functions. An example is the notable functions of the *DREB1* and *DREB2* genes, which are specifically activated in response to cold and drought stress [144]. *DREB* genes play several key roles in plants under drought stress. The function of *DREB* TFs is such that by binding to dehydration-responsive elements (DRE) in the promoter region of target genes, they regulate their expression [147]. These target genes usually include genes related to the production of osmolytes, antioxidant proteins, and other protective proteins that help plants perform better under drought stress [148]. Osmolytes, like proline and glycine betaine, are produced more abundantly when *DREB* genes are induced. These osmolytes aid in cellular osmotic homeostasis and protect cells from drought-induced damage. The modulation of abscisic acid (ABA) signaling pathways is mediated by *DREB* factors. Abscisic acid is recognized as a pivotal hormone in the context of drought stress, and *DREB* has a role in enhancing drought tolerance by controlling the expression of genes associated with this hormone [149]. *DREB* genes increase the expression of antioxidant-related genes, which helps to decrease oxidative damage produced by drought stress. These proteins have the ability to protect cells from free radical damage and aid in their maintenance [144].

DREB factors can affect root development. Because of this action, plants are able to produce deeper roots, which allows them to absorb more water even when the conditions are dry [150]. When it comes to signaling networks, *DREB* factors can create more intricate ones by interacting with other transcription factors like *NAC* and *MYB* [151]. These interactions can enhance the plant’s ability to cope with many types of stressors. Drought stress directly affects the expression of *DREB* genes. A decrease in soil moisture and an increase in temperature can lead to an increase in the expression of these genes, which can lead to the improvement of tolerance to drought and salinity in plants. Considering that DREB genes have a higher expression level in cultivars and species tolerant to drought stress, this parameter can be used as an indicator in the screening of lines tolerant to drought stress. A better understanding of the molecular mechanisms and the development of drought-resistant plants can be achieved through additional research into DREB genes, which are generally recognized as key regulators in plant responses to drought stress. Additionally, these genes can serve as an important target in stress-resistant plant genetic engineering. Table 1 lists some of the transcription factors involved in drought stress response in plants.

### 5.3. Stress-Responsive Genes

The induction of drought-stress-responsive genes is a complex cellular process that organisms, particularly plants, activate in response to the challenging conditions imposed by drought. The activation of stress-responsive genes typically involves intricate signaling pathways [164] and transcriptional regulation [165,166] under drought conditions. When a plant perceives the onset of drought, various signaling molecules are triggered, initiating a cascade of events that ultimately result in the induction of specific genes. These genes encode proteins with diverse functions, including the synthesis of osmoprotectants, antioxidants, and proteins that regulate water uptake and conservation. Overall, the activation of drought-stress-related genes is a dynamic and coordinated response that enhances a plant’s capability to tolerate and survive in periods of water scarcity. Understanding the molecular mechanisms behind this induction is crucial for developing crops with improved drought tolerance, an essential aspect in the face of changing climate patterns and increasing water scarcity.

**Late embryogenesis-abundant (LEA)** The LEA gene family was initially discovered in cotton, and it has subsequently been investigated in numerous other plants. It is considered to be one of the most significant gene families in plants and plays a major role in response to drought stress [167]. LEA1 to LEA6, dehydrin, and SMP are subtypes of LEA proteins with repetitive hydrophilic amino acid sequences [168]. LEA proteins engage a variety of ways to maintain water balance in cells during drought stress. LEA proteins possess hydrophilic features that enable them to retain water. This property keeps the cells from drying out by keeping moisture in. Under conditions of dehydration, these proteins can function as water reservoirs and assist in preserving osmotic equilibrium [169]. Another thing about LEA proteins is that they can form structures that look like gels when they do not have enough water. These structures perform the function of a physical barrier, preventing water from evaporating from the surface of the cells to the outside [170]. During the key stages of plant growth particularly during the embryonic stage, this feature is very important. Another example is the regulation of osmolytes, in which LEA proteins aid in the synthesis and accumulation of osmolytes such as proline [171]. These osmolytes protect cells from drought-stress-related damage and aid in preserving the osmotic balance within them. Cells can efficiently survive external stimuli by maintaining their osmotic balance. Chaperone function is a crucial property of LEA proteins because it prevents the accumulation of aberrant proteins. This feature facilitates the preservation of the proper protein conformation of proteins and their functionality in challenging circumstances [172]. Cells can adapt to external stimuli more efficiently if their proteins are functioning properly. The final aspect is the interaction of LEA proteins with the cell membrane, which assists in the stabilization of the membrane during dehydration. These interactions can limit membrane permeability and stop water loss in cells [168]. By utilizing these processes, LEA proteins can shield cells from harm caused by drought stress and improve plant performance in challenging environments.

**Dehydrins** Dehydrins, also known as Group II LEA proteins, are intrinsically disordered, highly hydrophilic proteins that help plants respond to abiotic conditions, including drought and excessive salinity [172]. These proteins play protective roles by stabilizing cellular structures with chaperone-like and detergent properties, with an array of cytoplasmic and nuclear targets, and maintaining essential cellular functions under adverse conditions [173,174]. The mechanisms of dehydration tolerance are not yet fully understood. Dehydration activates the synthesis of proteins involved in drought tolerance through both ABA-dependent and ABA-independent regulation. LEA proteins play a crucial role in enhancing drought, osmotic, or desiccation tolerance in plants. Dehydrins, the best-known LEA proteins, are ubiquitously expressed in plants during periods of low intracellular water content to increase their tolerance to desiccation [175].

Dehydrins exhibit high hydrophilicity and fulfill multifaceted functions in safeguarding plant cells under drought conditions. Evidence accumulated over the years indicates that dehydrin proteins confer drought tolerance by improving water retention capacity, preserving photosynthetic machinery, increasing chlorophyll content, promoting ROS detoxification, and facilitating the accumulation of compatible solutes [176]. On the other hand, are recognized as stress proteins that contribute to the formation of plants’ protective responses to dehydration. They can also be classified as hydrophilins.

Drought stress leads to dehydration and cell damage, triggering the induction of dehydrin genes. Eight dehydrins were found to respond to water-deficit stress, with the transcription quantity of specific dehydrins such as *PgDHN16*, *PgDHN10*, *PgDHN35*, and *PgDHN33* increasing significantly after a short period of water scarcity in white spruce [177]. Similarly, the *CdDHN4*, *YSK2*-type dehydrin was strongly up-regulated by drought in bermudagrass genotypes [178]. Furthermore, the accumulation patterns of dehydrins varied with the developmental stage during drought stress in plants, as observed in winter wheat at various growth stages [179]. The subcellular localization of dehydrins is consistent with their role as intracellular stabilizers, possibly with surfactant characteristics, acting upon targets in both the nucleus and cytoplasm [180]. Overall, the research on dehydrins and LEA proteins provides valuable insights into their roles in plant stress response, offering potential applications for the breeding of drought-tolerant cultivars in crops.

**Aquaporins** Aquaporins, situated in membrane channels, are essential for facilitating the transmembrane transport of water and maintaining osmotic and water homeostasis in plant cells [181]. Aquaporins are part of a highly conserved superfamily of membrane proteins known as major intrinsic proteins (MIP). Aquaporins can be categorized into five major subfamilies: tonoplast intrinsic proteins (TIPs), plasma membrane intrinsic proteins (PIPs), nodulin 26-like intrinsic proteins (NIPs), small and basic intrinsic proteins (SIPs), and x-intrinsic proteins (XIPs) [181]. Among these, TIPs and PIPs facilitate the primary vessels for intracellular water transport, regulate intercellular and intracellular water balance during stress, and play key roles in various aspects of adaptation to drought stress [182].

## 6. Phytohormone Regulation

There are a number of phytohormones that have an impact on how plants grow, develop, and react to environmental challenges like drought. These phytohormones include auxins, gibberellins, ethylene, ABA, and cytokinins [183]. Plant hormones and mitogen-activated protein kinases (MAPK) signaling pathways interact functionally, though the molecular mechanisms behind this cross-talk remain unclear [183]. These hormones act as signaling molecules, coordinating various physiological and molecular responses under water deficit conditions. Because of its many roles under environmental stress, especially drought, abscisic acid (ABA) is one of the most sought-after hormones for engineering crop plants to tolerate abiotic stress. It is recognized as a critical messenger that regulates the expression of stress-responsive genes and is involved in the stress-adaptive response of plants. Several enzymes in the ABA biosynthetic pathway have been manipulated to enhance abiotic stress tolerance in transgenic plants [184]. Here’s a summary of the role of ABA in drought tolerance signaling pathways:

### 6.1. Abscisic Acid (ABA)-Dependent Signaling

**Signaling cascade** ABA binding to its receptors leads to activating a signaling cascade. This cascade involves phosphorylation events and the regulation of key transcription factors, including ABFs (ABA-responsive element binding factors). ABFs facilitate the fast activation of group A *PP2C* genes by ABA, thereby contributing to the negative feedback regulation of ABA signaling [63]. ABI5 modulates seed germination via feedback regulation of the expression of the PYR/PYL/RCAR ABA receptor genes, revealing the regulatory function of ABI5 in ABA-facilitated germination of the seeds [185]. Furthermore, recent research has emphasized regulating the carbon/nitrogen (C/N) response mediated by a non-canonical ABA signaling pathway that operates independently of ABA biosynthesis. Additionally, it has revealed recent discoveries regarding the direct crosstalk between various cellular signals and the ABA signaling cascade [186]. Moreover, a study identified the *ARF2*-*ANT*-*COR15A* gene cascade as a signaling pathway mediated by ABA that connects the regulation of seed mass to water-deficit tolerance [187].

**Stomatal regulation** ABA has an important contribution to the modulating stomatal aperture. When water is limited, stomatal closure is induced by ABA, reducing water loss through transpiration and helping the plant conserve water. ABA regulates stomatal aperture through various signaling pathways and interactions with other molecules. For example, the OPEN STOMATA1 (OST1) protein kinase plays a key role in ABA-mediated guard cell signaling, and its mutants show impaired stomatal closure in response to ABA at various environmental stimuli [188]. Also, phytochrome interacting factors (PIFs) act as negative regulators of stomatal aperture by synchronizing ABA signaling pathways and red light [36]. Furthermore, ABA-induced stomatal closure can be modulated by other factors, such as 5-aminolevulinic acid (ALA) and ethylene, which act together with ABA signaling to control stomatal drive [189]. The regulation of stomatal movement by ABA entails a complex process that involves various molecular mechanisms and signaling pathways.

ABA signals are perceived by guard cells, leading to changes in ion fluxes and cell turgor pressure, resulting in stomatal closure. The search results confirm that ABA signals are indeed perceived by guard cells, leading to changes in ion fluxes and cell turgor pressure, resulting in stomatal closure. ABA is actively synthesized in the vascular tissues of both leaves and roots and then transported to guard cells. ABA is recognized by pyrabactin resistance (PYR)/PYR1-like (PYL)/regulatory factors of ABA receptor (RCAR) receptors, which deactivate PP2C, leading to the activation of the protein kinases SnRK2s. Many proteins involved in regulating stomatal closure are activated by SnRK2s through protein phosphorylation. ABA-activated SnRK2s stimulate the production of apoplastic ROS outside of guard cells, which are then transported into the guard cells. The apoplastic H_2_O_2_ can be directly detected by a receptor kinase known as HYDROGEN PEROXIDE-INDUCED Ca^2+^ INCREASES1. Calcium ions (Ca^2+^) and ROS also contribute to controlling stomatal closure [76]. Additionally, phosphatidic acid (PA) and microtubules are also associated with ABA-induced stomatal closure [190,191]. Nitrate reductases NIA1 and NIA2 are critical to ABA-induced stomatal closure, and their loss disrupts stomatal closure by modifying genes encoding core ABA signaling factors in Arabidopsis [192]. Overall, ABA signals are perceived by guard cells, resulting in changes in ion fluxes and cell turgor pressure, resulting in stomatal closure through various signaling pathways and interactions with other molecules.

**Osmotic adjustment** Osmotic stress induces the synthesis of osmoprotectants and compatible solutes such as several disaccharides (trehalose, sucrose, maltose), glycine betaine, and proline. In response to desiccation, these organic molecules accumulate in cells contributing to osmotic adjustment due to their highly hydrophilic properties. Osmoprotectants, also known as compatible solutes or osmolytes, help maintain cellular turgor and prevent water loss from cells. Among phytohormone-mediated signaling pathways, ABA-induced osmotic adjustment is a prime drought stress adaptive mechanism that supports plant production by maintaining turgor and protecting cellular functions [50]. Alongside this, the ABA has a crucial contribution to plant adaptation to osmotic stress by regulating the synthesis of osmoprotectants. ABA enhances the synthesis of glycine betaine, an important compatible solute that protects cells from osmotic stress caused by dehydrating conditions [193]. Also, ABA is involved in the dynamic adjustment of sugar and proline concentrations within plant cells, which are vital for tolerance to environmental stress [194]. Therefore, ABA has a crucial contribution in regulating the synthesis of osmoprotectants and compatible solutes, contributing to tolerance to osmotic stress and the maintenance of cellular turgor in plants.

**Interaction of ABA and other phytohormones** In plants, the ABA hormone interacts with other phytohormones, creating a complex network of signaling pathways. For example, ABA interacts with ethylene, jasmonic acid, and salicylic acid, coordinating responses to various stresses. Light signals modulate ABA signaling, and light photoreceptors (i.e., phyA and phyB) negatively regulate ABA signaling, while the photomorphogenic central repressor COP1 positively regulates ABA signaling in yeast cells [101]. Additionally, the interaction between ABA and light significantly contributes to plant responses to drought. There is evidence of the integration of drought- and light-induced ABA signaling pathways to combat drought stress in plants [195]. Furthermore, in Arabidopsis, ABA interacts with NIA1 and NIA2 known as nitrate reductases, as their loss interferes with stomatal closure by modifying genes associated with core ABA signaling factors [192].

**Root architecture and water uptake** ABA influences root architecture, promoting the growth of deeper roots. This allows plants to explore soil layers with higher water availability, enhancing water uptake efficiency. A study on rice reported that ABA has a crucial contribution to regulating root development and drought resistance, indicating that ABA influences root architecture to enhance plant resilience to water scarcity [196]. Research in Arabidopsis demonstrated that the *Pseudomonas argentinensis*, a beneficial root endophytic bacterium, improves water-deficit tolerance in plants by mediating gene expression and root morphogenesis via the ABA pathway, further pinpointing the role of ABA in promoting tolerance through root architecture modifications [197].

**Seed dormancy and germination** ABA regulates seed dormancy and germination. During drought periods, ABA maintains seed dormancy to prevent premature germination in unfavorable conditions. ABA is a pivotal hormone that induces dormancy during seed development on the mother plant and, after seed dispersal, regulates dormancy release and germination in response to environmental cues [198]. ABA accumulation and signaling contribute to the response of plants to water-deficit stress, regulating the alterations in root system architecture, stomatal dynamics, and the timing of senescence to protect against stress [195]. Moreover, ABA governs the subcellular relocation of OsABI-LIKE2, a negative regulator in ABA signaling, to modulate root architecture and, in turn, enhance tolerance to drought stress in rice [196].

### 6.2. Abscisic Acid (ABA)-Independent Signaling

The term “ABA-independent signaling” refers to a wide range of pathways that do not rely on ABA for their function. Reactive oxygen species (ROS), calcium ions (Ca^2+^), and other phytohormones like ethylene and jasmonic acid are involved in the signaling processes that comprise these pathways [199]. Signaling pathways that are not dependent on ABA include calcium signaling, RLK signaling, and MAPK signaling, which was previously described. They serve critical roles in the plant’s ability to perceive environmental stress signals and activate downstream responses, which ultimately contribute to the plant’s overall adaptability to drought stress. This contrast emphasizes the intricacy of plant signaling networks, in which both ABA-dependent and ABA-independent pathways collaborate to improve drought responses.

## 7. Crop Improvement for Drought Tolerance

Breeding and genetic modification have long been used to develop drought-tolerant crops. Recent advances in molecular biology and genetic engineering have introduced new tools for the development of resilient cultivars. Traditionally, introduction, selection, and hybridization techniques have been employed to improve crop drought tolerance. However, modern tools such as molecular markers and genetic engineering now offer enhanced efficiency and speed in breeding methods. The availability of reliable QTLs that account for a significant portion of the genetic variance in drought tolerance will enable breeders to select plants with improved resistance to water deficit, even at early growth stages [200,201]. Transgenic-based genetic engineering offers an alternative to traditional back-crossing methods for introducing drought-tolerance genes. This technology has the advantage of bypassing hybridization barriers, enabling gene transfer from any organism into a plant variety. Genome editing can further refine desirable traits by targeting specific genes, such as knocking out malfunctioning alleles that negatively affect drought tolerance or correcting mutant alleles responsible for traits like water-use efficiency [201,202]. Ultimately, the combination of classical breeding and modern molecular techniques will accelerate the development of crop cultivars with enhanced tolerance to drought stress.

## 8. Challenges and Future Perspectives

Globally, and especially in arid and semi-arid environments, plants inevitably confront water scarcity, posing a significant threat to their growth and overall productivity. The search results provide insights into the challenges and future directions related to plant adaptation to water scarcity, particularly in semi-arid and arid environments.

The findings emphasize the importance of expressing cellular compatible solutes, protective proteins, polyphenols, terpenoids, and enzymatic antioxidants, which are crucial to adapting to drought stress. While there has been some skepticism about the role of osmotic adjustment in sustaining crop yield under drought stress, a critical review of published studies revealed a positive and significant association between osmotic adjustment and yield under drought stress in various crops. In addition, incorporating ABA and light signaling pathways has been identified as a critical strategy for combating drought stress in plants. ABA signaling and accumulation play a vital role in regulating physiological responses, such as root system architecture, stomatal dynamics, induction of protective proteins, and senescence, to counter the adverse effects of drought. Drought tolerance is a multifaceted trait governed by many genes involved in the networks of various adaptive mechanisms. Given that plant breeders work within such a continuum of trait genetic complexity, breeding strategies such as applying stable QTLs in selection, gene pyramiding, transgene pyramiding, and transgenic RNAi would be viable alternatives. Future transgenic solutions for this complex trait will necessitate the introduction of multiple genes, either to confer stress tolerance or to incorporate several genes within a pathway. In our view, plant physiology also faces truly grand challenges of understanding how drought affects plants and how drought-tolerant plants function and survive under water stress, while providing at least partial solutions to the critical needs of breeders. Achieving these goals will require a multidisciplinary approach that includes molecular engineering, with plant physiology serving as a foundational component.

## Figures and Tables

**Table 1:** Summary of the transcription factors involved in drought response in plants.

Gene Name	Plant Species	Functional Description	Reference
*TwNAC01*	Arabidopsis and Triticale	Positively regulates drought stress responses	[130]
*SlNAC6*	(*Solanum lycopersicum* L.)	Involved in drought stress response and reproductive process in tomato	[152]
*RcNAC72*	* Rosa chinensis * Jacq.	*RcNAC72* is recognized by *RcABF4*, interacts with *RcDREB2A* to enhance drought tolerance in Arabidopsis	[153]
*LpNAC17*	* Lilium pumilum * L.	Stress-responsive *NAC* transcription factor *LpNAC17* enhances salt stress tolerance in tobacco	[130]
*GhNAC072*	* Gossypium hirsutum * L.	Overexpression of cotton *GhNAC072* gene enhances drought and salt stress tolerance in transgenic Arabidopsis	[154]
*MdNAC29*	apple ( *Malus domestica* L.)	The *NAC* transcription factor *MdNAC29* negatively regulates drought tolerance in apple	[101]
*PagSAP11*	hybrid poplar ( * Populus alba * × * Populus tremula * var. glandulosa)	Knockdown of *PagSAP11* confers drought resistance and promotes lateral shoot growth in hybrid poplar	[155]
*SlERF84*	* S. lycopersicum *	A tomato *ERF* transcription factor, *SlERF84*, confers enhanced tolerance to drought and salt stress but negatively regulates immunity against *Pseudomonas syringae* pv. tomato DC3000	[156]
*BrERF109*	Chinese cabbage (*Brassica rapa* L. ssp. *pekinensis*)	Silencing of *BrERF109* in Chinese cabbage by virus-induced gene silencing (VIGS) led to plants’ susceptibility to drought and salt stress	[157]
*TaERF87*	* Triticum aestivum * L.	*TaERF87* and *TaAKS1* synergistically regulate *TaP5CS1*/*TaP5CR1*-mediated proline biosynthesis to enhance drought tolerance in wheat	[158]
*DcAP2/ERF96*	*Dendrobium catenatum* Lindley	Genome-wide identification of *AP2/ERF* transcription factor family and functional analysis of *DcAP2*/*ERF*#96 *Associated with Abiotic Stress* in *Dendrobium catenatum*	[153]
*TaERF3*	* Triticum aestivum * L.	The *ERF* transcription factor *TaERF3* promotes tolerance to salt and drought stresses in wheat	[159]
*DREB2A*	* Picea wilsonii * Mast	*Picea wilsonii NAC31* and *DREB2A* Cooperatively Activate *ERD1* to modulate drought resistance in transgenic Arabidopsis	[132]
*TaPP2C158*	* Triticum aestivum * L.	DIW1 encoding a clade I PP2C phosphatase negatively regulates drought tolerance by de-phosphorylating TaSnRK1.1 in wheat	[158]
*RcMYB8*	rose ( * Rosa chinensis Jacq *.)	*RcMYB8* enhances salt and drought tolerance in rose (*R. chinensis*) by modulating *RcPR5*/*1* and *RcP5CS1*	[150]
*TaTIP41 and TaTAP46*	* Triticum aestivum * L.	*TaTIP41* and *TaTAP46* positively regulate drought tolerance in wheat by inhibiting *PP2A* activity	[147]
*MuWRKY3*	* Macrotyloma uniflorum * Lam. Verdc	A novel WRKY transcription factor, MuWRKY3 (*Macrotyloma uniflorum* Lam. Verdc.) enhances drought stress tolerance in transgenic groundnut (*Arachis hypogaea* L.) Plants	[160]
*PoWRKY71*	* Paeonia ostii *	*PoWRKY71* is involved in *Paeonia ostii* resistance to drought stress by directly regulating light-harvesting chlorophyll a/b-binding 151 gene	[161]
*OsWRKY97*	* Oryza sativa * L.	*OsWRKY97*, an abiotic stress-induced gene of rice, plays a key role in drought tolerance	[37]
*29A and RD29B*	* Arabidopsis thaliana *	*RD29A* and *RD29B* rearrange genetic and epigenetic markers in priming systemic defense responses against drought and salinity	[142]
*VyUSPA3*	* Vitis yeshanensis *	*VyUSPA3*, a universal stress protein from the Chinese wild grape Vitis yeshanensis, confers drought tolerance to transgenic V. vinifera	[162]
*MtCBL13*	* Medicago truncatula * L.	*MtCBL13* confers drought sensitivity in Arabidopsis through ABA-dependent pathway	[163]

## Data Availability

Data are contained within the article.
